# Evaluation of Osteopontin Gene Expression in Endometrium of Diabetic Rat Models Treated with Metformin and Pioglitazone 

**DOI:** 10.22074/ijfs.2019.5471

**Published:** 2018-10-02

**Authors:** Zeinab Sadat Hosseiny, Parvaneh Nikpour, Abas Bakhtiary, Fatemah Sadat Mostafavi, Mohammad Matinfar, Mehrnaz Jahani, Roshanak Aboutorabi

**Affiliations:** 1 Department of Anatomical Sciences, Faculty of Medicine, Isfahan University of Medical Sciences, Isfahan, Iran; 2 Department of Genetics and Molecular Biology, Faculty of Medicine, Isfahan University of Medical Sciences, Isfahan, Iran; 3 Department of Internal Medicine, Faculty of Medicine, Isfahan University of Medical Sciences, Isfahan, Iran

**Keywords:** Diabetes, Endometrium, Implantation, Osteopontin

## Abstract

**Background:**

Osteopontin (*Opn*) is one of the co-factors involved in cell adhesion and invasion during the implanta- tion process. Several reports have shown *Opn* expression changes in diabetic condition in several tissues. In addition, an increased incidence of spontaneous abortion is reported in diabetic women. We, therefore, designed a study to eval- uate the effects of diabetes on *Opn* expression at implantation time after treatment with metformin and pioglitazone.

**Materials and Methods:**

In this interventional and experimental study, 28 rats were randomly divided into four groups, namely control, diabetic, pioglitazone-treated diabetic rats and metformin-treated diabetic rats. Streptozo- tocin (STZ) and nicotinamide (NA) were used to induce type 2 diabetes (T2D). During the implantation window, the endometrium was removed and the expression of *Opn* was analysed by reverse transcription quantitative polymerase chain reaction (RT-qPCR).

**Results:**

*Opn* expression was significantly higher (30.70 fold-changes) in the diabetic group in comparison with the control group (P=0.04). Furthermore, the expression of *Opn* was significantly lower in the diabetic group treated with pioglitazone when compared with the diabetic group (P=0.04).

**Conclusion:**

According to the high *Opn* expression and the possibility of increased adhesion of endometrial epithelial cells, the invasion of blastocyst may be affected and thus reduced. As pioglitazone significantly reversed the upregula- tion of *Opn* in diabetic rats, it may be considered as a therapeutic compound for treating T2D.

## Introduction

Globally, approximately 60 to 80 million couples are likely to be affected by infertility per year (1). About 15% of couples suffer from subfertility around the world (2). The rate of male and female infertility incidences have been estimated to be equal with each explaining approximately 35-40% of cases. The cause of infertility in the remaining proportion of cases (male or female) may be due to a combination of disorders in the two genders (20- 30%) (3), through which, 15-30% of cases are diagnosed as unexplained infertility (4).

The first step to achieve a successful pregnancy is embryo implantation which needs an intact embryo, an endometrium and the synchronization between them (5). Failure in implantation is multifactorial and may be due to embryonic or maternal dysfunction during the dialogue window between them. Coagulation, immunological, endothelial, endocrine and metabolic disorders are among the most common known causative factors of subfertility (6, 7).

Diabetes is a variety of metabolic diseases in which individuals are unable to produce or uptake adequate levels of insulin, resulting in high levels of blood glucose (8). Diabetes mellitus could deregulate a variety of cellular and molecular pathways (9).

A total of 425 million diabetic individuals have been reported worldwide in 2017 and it is estimated that this population will reach 629 million by 2045 (10). The incidence of type 2 diabetes (T2D) is also rising and is accompanied with age reduction in its onset, especially in women (11).

Diabetes can seriously affect the outcome of embryo implantation and pregnancy. It seems diabetes mellitus impairs the molecular functions of the female reproductive system and thus causes improper implantation and/or fetal loss (12).

Some reports have shown that miscarriage, neonatal morbidity and mortality, and neonatal congenital malformations are observed in women who suffer from T2D (13, 14).

Administration of metformin to T2D patients for blood glucose level reduction is common. Metformin affects cell insulin resistance, descends gluconeogenesis by liver and increases blood glucose utilization, therefore leading to euglycemia (15).

Pioglitazone is a member of the thiazolidinediones (TZDs) family, which is used as an antidiabetic drug. It acts by binding to peroxisome proliferator-activated receptor gamma (PPAR-γ). This drug therefore improves glycemic control by increasing insulin sensitivity at cellular level (16).

The association of subfertility or infertility with diabetes, as a metabolic disease, has been previously evaluated (17). However, the effect of diabetes on gene expression at the transcript and protein levels have not been evaluated during the implantation window (14).

The embryo-maternal crosstalk during the implantation window involves several genes which ought to be expressed at the right time either in the blastocyst or the endometrium (18). Receptivity of endometrium is the key point for implantation of the blastocyst (18, 19). This receptivity is provided by a number of molecules which which reach their peak values during the window of implantation (5). Some of these molecules include integrins, mucins, vascular endothelial growth factor (VEGF), and osteopontin (20). Integrin family members act as receptors for multiple ligands such as osteopontin, laminin and collagen.

In rats, *Opn* gene has 7 exons, and its location is on the 14p22) (21). Osteopontin (*Opn*) promotes cell adhesion and invasion through its Arg-Gly-Asp domain (5). *Opn* is shown to be present at a high level in the epithelial layer (during mid-secretary phase) in human, mouse and rabbit uterine (22, 23). Osteopontin has been also identified as a protein associated with metastatic cancers, as an extracellular matrix protein of bones and teeth, as a cytokine produced by activated lymphocytes and macrophages, and as a major constituent of the uterus and placenta during pregnancy (22).

Given the rise of T2D prevalence its effects on the female reproductive system, we quantified the expression of *Opn* in the endometrium of diabetic rat models to examine the association of *Opn* with T2D and evaluate the molecular effect of metformin and pioglitazone treatments on *Opn* expression.

## Materials and Methods

### Animal and maintenance

This interventional and experimental study on diabetic rat models was conducted at the Central Laboratory of Isfahan University of Medical Sciences in 2017. This work has the Ethical Committee code number IR.MUI.REC.1394.1.184.

Adult virgin female Wistar rats weighting 200-250 g were obtained from Pasteur Institute of Iran, aged 6-8 weeks, maintained in conventional wire mesh cages at room temperature 21 ± 1°C and humidity of 45-50% with light/dark cycle. Rats had access to standard dry pellets and water.

### Induction of diabetes

Diabetes was induced in rats by injecting 60 mg/kg streptozotocin (STZ, Sigma-Aldrich Chemie, Germany) intraperitoneally. Fifteen minutes prior to STZ injection, 200-230 mg/kg nicotinamide (NA, Sigma-Aldrich Chemie, Germany) was injected intraperitoneally (24-26).

Blood samples were taken from the tail vein and glucose level values were measured using a glucometer (HemoCue Glucose 201+, Ängelholm, Sweden). Rats with blood glucose levels above 250 mg/dl were considered manifestly as diabetic (27).

### Study design and tissue collection

The 28 rats were randomly categorized into four groups (n=7), namely control, diabetic, Pioglitazone-treated and metformin-treated diabetic rats.

The first group of rats was the control group and did not receive any substance. The second (diabetic) group did not receive any treatment except for STZ and NA. The third group received 20 mg/kg/day of pioglitazone for diabetes treatment (28), and the final group received 100 mg/kg/day of metformin (12). Treatments were administered by orogastric gavage and continued for 4 weeks (28).

Rats were maintained in diabetic condition for 3 weeks (one sexual cycle) and then underwent treatment with metformin or pioglitazone.

Treatments with the two drugs lasted 4 weeks and for the next step, each of the 3 female rats were mated with 1 male rat and vaginal plug was observed the following morning (first day of pregnancy). Animals were anesthetized and sacrificed on the 4^th^ day of pregnancy, considered as the implantation day (6). The rat uteri were then removed, snap-frozen in liquid nitrogen and stored at -80°C for further analysis

### Real time polymerase chain reaction

#### RNA extraction

Total RNA was isolated from epithelial cells of endometrium using the RNX plus solution (Cinnagen, Iran) according to the manufacturer’s instructions and as previously described. The purity and integrity of the extracted RNA were assessed by optical density measurements (260/280 nm ratios) and by visual observation of samples electrophoresed on agarose gels. For elimination of genomic DNA, RNA was treated with RNase-free DNase (Qiagen, Germany)

#### cDNA synthesis

Complementary DNA (cDNA) synthesis was carried out by using a cDNA synthesis Kit (Yektatajhiz, Iran). Briefly, the synthesis mixture was prepared by adding 4 μl of 5 X first-strand buffer, 1 μl of dNTPs, 0.5 μl of RNasin and 1 μl of M-MLV. Approximately 1 μg of RNA and random hexamer primers were finally added to the mixture in a 20 μl reaction

### Quantitative real-time polymerase chain reaction

Specific primers for the rat β-actin (as an internal control, Accession number: NM_031144) and osteopontin (NM_012881.2) genes were designed with Genrunner software version 3.05 (Hastings Software, Hastings, NY, USA). All designed primers were checked against the the rat genome using BLAST to make sure they are not complementary with other regions of genome.

The sequences of the designed primers are as follow:

SPP1- F: 5´-AGGAGAAGGCGCATTACAG-3´
R: 5´-GCTTTCATTGGAGTTGCTTG -3´

with an amplicon size of 160 bp and

β-actin- F: 5´-GCCTTCCTTCCTGGGTATG-3´
R: 5´-AGGAGCCAGGGCAGTAATC-3´

with an amplicon size of 165 bp.

PCR was carried out by using the specific primers along with the Maxima™ SYBR Green/ROX qPCR Master MIX (Fermentas, Lithuaria) and run on an Applied Biosystems StepOnePlus instrument. The PCR cycling conditions were an initial denaturation step at 95˚C for 10 minutes, followed by 40 amplification cycles of denaturation at 95˚C for 10 seconds, annealing at 60˚C and 58.8˚C for β-actin and osteopontin genes respectively for 10 seconds, and extension at 72˚C for 10 seconds. All samples were measured in duplicate. The 2^-ΔΔCt^ method was utilized to quantify the relative levels of gene expression.

### Statistical analyses

Statistical analyses were performed using SPSS version 18.0 (SPSS Inc, Chicago, IL, USA). All data are expressed as mean ± standard error of mean (SEM) from at least in triplicate at two separate experiments. Differences between groups were analyzed using Analysis of Variance (ANOVA) with post hoc multiple comparisons. Statistical significance was defined as P<0.05.

## Results

**Fig.1:**
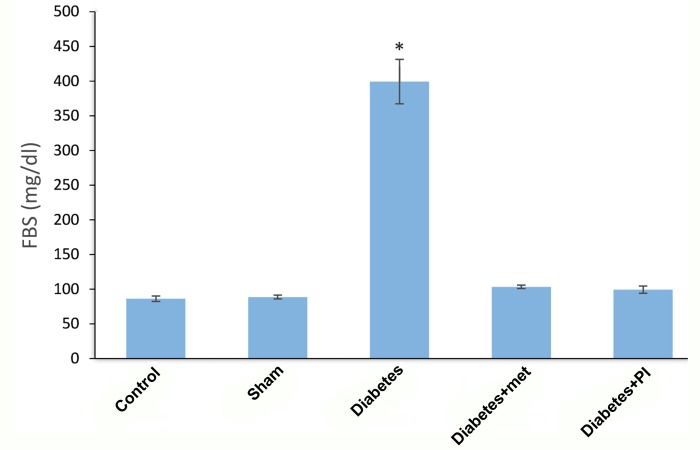
Fasting plasma glucose concentrations in diabetic rat models and in those treated with metformin and pioglitazone. *; Significant difference between the diabetic group and other groups (P=0.0001), FBS; Fasting blood sugar, met; Metformin, and PI; Pioglitazone.

Figure 1 shows fasting plasma glucose concentrations in diabetic rat models (399.28 ± 84.61) and in those treated with metformin (103.28 ± 14.12) and pioglitazone (99.29 ± 6.70). There was a significant difference between the diabetic group and all other groups (P=0.0001).

**Fig.2:**
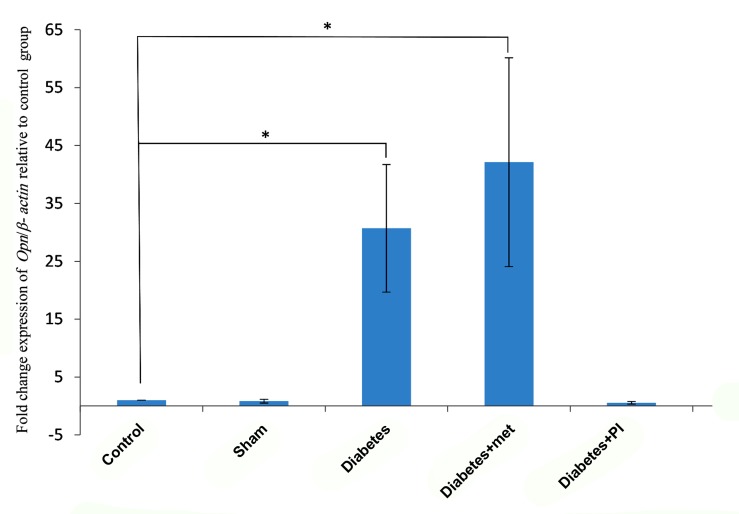
Fold change expression of *Opn* /β-actin gene relative to the control group based on real time polymerase chain reaction analysis in the rat endometrium. *Opn* transcript expression was significantly higher in the diabetic and diabetic+met groups in comparison with the control subjects (P=0.04). *; P<0.05, met; Metformin, and PI; Pioglitazone.

The differential expression of the target gene was compared with the house keeping gene (β-actin) in all samples. As shown in Table 1 and Figure 2, the mean of *Opn* expression in the diabetic group (30.70 ± 11.65) was significantly different from the control group (1) (P=0.04) but no significant difference was observed between diabetic and metformin treated group (42.11 ± 19.07) (P=0.07). Therefore in diabetic group and diabetic treated with metformin group upregulation in expression of *Opn* gene were observed. Also, the diabetic treated with pioglitazone group (0.55 ± 0.22) showed no significant difference compared with the control group (1) (P=0.3).

**Fig.3:**
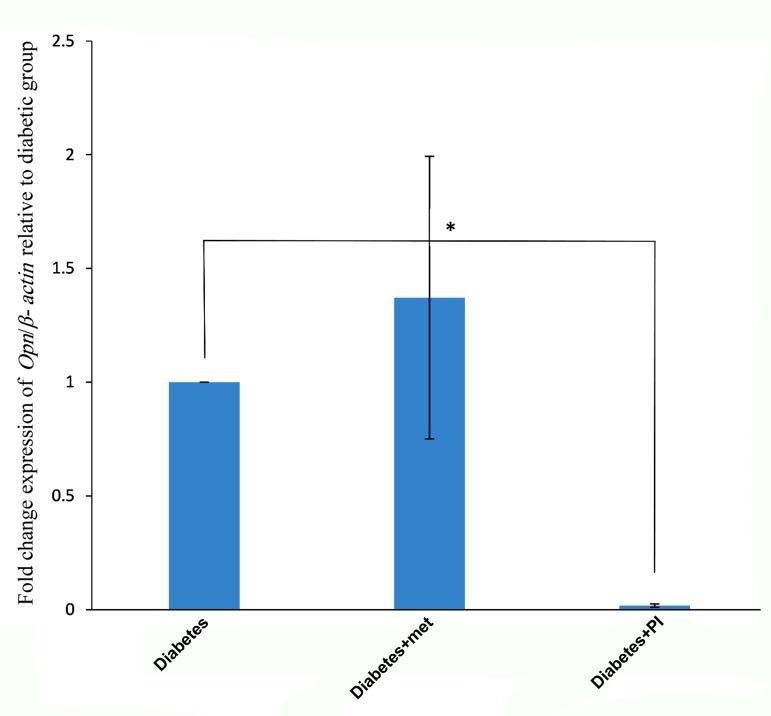
Fold change expression of *Opn*/β-actin gene relative to the diabetic group. *Opn* mRNA expression was significantly lower in the diabetic group treated with pioglitazone (P=0.04). *; P<0.05, met; Metformin, and PI; Pioglitazone.

Figure 3 shows the non-significant difference observed in *Opn* mRNA expression in metformin treated group (1.371 ± 0.621) compared to diabetic group (1) (P=0.62). On the other hand, there was a significant reduction in the expression of *Opn* in pioglitazone treated group (0.017 ± 0.007) compared with the diabetic group (1) (P=0.04).

**Table 1:** Mean level of Opn transcript expression in different groups


Groups	Specific (mean ± SEM)	Normalized (mean ± SEM)

Control	0.35	1
Sham	0.29 ± 0.12	0.83 ± 0.35
Diabetic	10.99 ± 4.17	30.70 ± 11.65
Pioglitazone treated	0.19 ± 0.08	0.55 ± 0.22
Metformin treated	15.08 ± 6.83	42.11 ± 19.07


## Discussion

This study was designed to investigate the effects of diabetes on *Opn* gene expression at the implantation stage after being treated with metformin and pioglitazone. Osteopontin transcript expression was significantly higher in the diabetic group in comparison with the control group. Furthermore, evidence for the ability of pioglitazone to downregulate *Opn* expression was shown. Given the high *Opn* expression and the possibility of increased adhesion of endometrial epithelial cells, the invasion of blastocyst may be affected and thus reduced. As pioglitazone significantly reverted the *Opn* expression in diabetic rats, it may be considered as a therapeutic compound to act against this molecular perturbation.

Receptivity of endometrium, mature blastocyst and dialogue between them are essential for the multifactorial nature of embryo implantation. The duration of this dialogue window is different among mammals, but should be present for a limited time for embryo reception (5).

Failure in the onset of pregnancy is widely due to inappropriate endometrial receptivity (29). Several endometrial growth factors, cytokines and adhesion molecules such as osteopontin cooperate in molecular pathways which are necessary for pregnancy (30, 31).

*Opn* transcript level increases at the implantation sites from day 5 to 8 of pregnancy in the mouse uterus. Therefore, *Opn* expression is thought to be essential for hatching and adhering the trophoblast to the endometrium (5).

In addition, at the protein expression level, *Opn* and β3 integrin positive cells were significantly higher on the 5th day of pregnancy. The presence of these two proteins were proposed as suitable markers for predicting the fate of ongoing implantation by the authors (32). *Opn* expression is also reported during the peri-implantation period, which is under control of progesterone in rabbit (33).

Young et al. (34) showed that in the proliferative phase of the menstrual cycle, *Opn* was not observed, however, its presence was observed during the secretory phase.

*Opn* expression has also been studied in normal endometrium during implantation in human (32), rat (23), mouse (35), sheep and pig (36). Here, we evaluated its expression in diabetic rats during the implantation window and observed significant overexpression when compared with the control group.

Diabetes mellitus in women could cause reduction of fertility, poor reproduction outcome and molecular abnormalities in ovary and endometrium (37). In diabetic mice models, the implantation outcome is shown to be lower than control mice (14).

Takemoto et al. (38) observed enhanced *Opn* expression levels in cultured rat aortic smooth muscle cells which were maintained in a medium with high glucose levels and suggested that it may be involved in the development of diabetic vascular complication.

Streptozotocin-induced diabetes mellitus in rats leads to the reduction of endometrial thickness while treating with pioglitazone and zinc improves the damages in the endometrium (25).

Up-regulation of *Opn* has been reported in renal tissue of diabetic rat models, which may implicate this molecule as a potential key pathophysiologic factor in diabetic nephropathy. Treatment with pioglitazone is thought to suppress *Opn* expression levels (39).

Consistently, we observed a significant reduction in the expression of *Opn* in the group treated with pioglitazone in comparison with the diabetic group. This indicates that pioglitazone has the ability to control the overexpression of *Opn* gene, which is probably in relation to the effect of pioglitazone on PPAR- γ (a regulator of gene expression). Further studies are nevertheless required to suggest administration of pioglitazone.

Another common drug for the treatment of diabetes is metformin, which causes an increase in intracellular magnesium concentration along with a lower blood glucose level in the uterus and ovary (12). Metformin seems to have positive effects on other organs such as the kidney where it significantly protects renal function in diabetic nephropathy (40). In this study, we observed no significant effect of metformin on *Opn* expression in the endometrium of diabetic rats.

## Conclusion

We conclude that due to the high expression of *Opn* and the possibility of increased adhesion of endometrial cells to each other, the invasion of blastocyst into uterine epithelium is likely reduced. Also, pioglitazone significantly down-regulates the expression *Opn* back to its normal levels in the female diabetic rats.

## References

[1] Sudha G, Reddy KSN (2013). Causes of female infertility: a crosssectional study. International Journal of Latest Research in Science and Technology.

[2] Agarwal A, Mulgund A, Hamada A, Chyatte MR (2015). A unique view on male infertility around the globe. Reprod Biol Endocrinol.

[3] Deyhoul N, Mohamaddoost T, Hosseini M (2017). Infertility-related risk factors: a systematic review. Int J Womens Health Reprod Sci.

[4] Quaas A, Dokras A (2008). Diagnosis and treatment of unexplained infertility. Rev Obstet Gynecol.

[5] Qi QR, Xie QZ, Liu XL, Zhou Y (2014). Osteopontin is expressed in the mouse uterus during early pregnancy and promotes mouse blastocyst attachment and invasion in vitro. PLoS One.

[6] Aplin JD, Kimber SJ (2004). Trophoblast-uterine interactions at implantation. Reprod Biol Endocrinol.

[7] Tuckerman EM, Laird SM, Prakash A, Li TC (2006). Expression of integrins in the endometrium of women with recurrent miscarriage. Fertil Steril.

[8] Beck JK, Cogen FR (2015). Outpatient management of pediatric type 1 diabetes. J Pediatr Pharmacol Ther.

[9] Saeedi Borujeni MJ, Esfandiary E, Taheripak G, Codoñer-Franch P, Alonso-Iglesias E, Mirzaei H (2018). Molecular aspects of diabetes mellitus: resistin, MicroRNA and exosome. J Cell Biochem.

[10] Kim ES, Jeong JS, Han K, Kim MK, Lee SH, Park YM (2018). Impact of weight changes on the incidence of diabetes mellitus: a Korean nationwide cohort study. Sci Rep.

[11] Feig DS, Murphy K, Asztalos E, Tomlinson G, Sanchez J, Zinman B (2016). Metformin in women with type 2 diabetes in pregnancy (MiTy): a multi-center randomized controlled trial. BMC Pregnancy Childbirth.

[12] Gales C, Zamfir C, Radulescu D, Stoica B, Nechifor M (2014). Protective effect of magnesium and metformin on endometrium and ovary in experimental diabetes mellitus. Magnes Res.

[13] Greene MF (1999). Spontaneous abortions and major malformations in women with diabetes mellitus. Semin Reprod Endocrinol.

[14] Wang TS, Gao F, Qi QR, Qin FN, Zuo RJ, Li ZL (2015). Dysregulated LIF-STAT3 pathway is responsible for impaired embryo implantation in a Streptozotocin-induced diabetic mouse model. Biology Open.

[15] Ainuddin JA, Karim N, Zaheer S, Ali SS, Hasan AA (2015). Metformin treatment in type 2 diabetes in pregnancy: an active controlled, parallel-group, randomized, open label study in patients with type 2 diabetes in pregnancy. J Diabetes Res.

[16] Filipova EP, Uzunova KH, Vekov TY (2015). Comparative analysis of therapeutic efficiency and costs (experience in Bulgaria) of oral antidiabetic therapies based on glitazones and gliptins. Diabetol Metab Syndr.

[17] Basmatzou T, Konstantinos Hatziveis M (2016). Diabetes mellitus and influences on human fertility. International Journal of Caring Sciences.

[18] Altmäe S, Martínez-Conejero JA, Salumets A, Simón C, Horcajadas JA, Stavreus-Evers A (2010). Endometrial gene expression analysis at the time of embryo implantation in women with unexplained infertility. Mol Hum Reprod.

[19] Boivin J, Bunting L, Collins JA, Nygren KG (2007). International estimates of infertility prevalence and treatment-seeking: potential need and demand for infertility medical care. Hum Reprod.

[20] Liu JL, Zhao M, Peng Y, Fu YS (2016). Identification of gene expression changes in rabbit uterus during embryo implantation. Genomics.

[21] Homepage N (2018). spp1 rat gene.

[22] Johnson GA, Burghardt RC, Bazer FW (2014). Osteopontin: a leading candidate adhesion molecule for implantation in pigs and sheep. J Anim Sci Biotechnol.

[23] Kang YJ, Forbes K, Carver J, Aplin JD (2014). The role of the osteopontin-integrin αvβ3 interaction at implantation: functional analysis using three different in vitro models. Hum Reprod.

[24] Rossetti L, DeFronzo RA, Gherzi R, Stein P, Andraghetti G, Falzetti G (1990). Effect of metformin treatment on insulin action in diabetic rats: in vivo and in vitro correlations. Metabolism.

[25] Gales C, Zamfir C, Stoica B, Nechifor M (2015). Zinc and pioglitazone effects on ovaries and endometrium in diabetes. Farmacia.

[26] Masiello P, Broca C, Gross R, Roye M, Manteghetti M, Hillaire-Buys D (1998). Experimental NIDDM: development of a new model in adult rats administered streptozotocin and nicotinamide. Diabetes.

[27] Zabihi S, Wentzel P, Eriksson UJ (2008). Altered uterine perfusion is involved in fetal outcome of diabetic rats. Placenta.

[28] Eissa LA, Abdel-Rahman N, Eraky SM (2015). Effects of omega-3 fatty acids and pioglitazone combination on insulin resistance through fibroblast growth factor 21 in type 2 diabetes mellitus. Egyptian Journal of Basic and Applied Sciences.

[29] Macklon NS, Stouffer RL, Giudice LC, Fauser BC (2006). The science behind 25 years of ovarian stimulation for in vitro fertilization. Endocr Rev.

[30] Aghajanova L, Hamilton AE, Giudice LC (2008). Uterine receptivity to human embryonic implantation: histology, biomarkers, and transcriptomics. Semin Cell Dev Biol.

[31] Gong X, Tong Q, Chen Z, Zhang Y, Xu C, Jin Z (2015). Microvascular density and vascular endothelial growth factor and osteopontin expression during the implantation window in a controlled ovarian hyperstimulation rat model. Exp Ther Med.

[32] Liu N, Zhou C, Chen Y, Zhao J (2013). The involvement of osteopontin and β3 integrin in implantation and endometrial receptivity in an early mouse pregnancy model. Eur J Obstet Gynecol Reprod Biol.

[33] Apparao KB, Illera MJ, Beyler SA, Olson GE, Osteen KG, Corjay MH (2003). Regulated expression of osteopontin in the peri-implantation rabbit uterus. Biol Reprod.

[34] Young MF, Kerr JM, Termine JD, Wewer UM, Wang MG, McBride OW (1990). cDNA cloning, mRNA distribution and heterogeneity, chromosomal location, and RFLP analysis of human osteopontin (OPN). Genomics.

[35] von Wolff M, Strowitzki T, Becker V, Zepf C, Tabibzadeh S, Thaler CJ (2001). Endometrial osteopontin, a ligand of β 3-integrin, is maximally expressed around the time of the “implantation window”. Fertil Steril.

[36] Girotti M, Zingg HH (2003). Gene expression profiling of rat uterus at different stages of parturition. Endocrinology.

[37] Cardozo E, Pavone ME, Hirshfeld-Cytron JE (2011). Metabolic syndrome and oocyte quality. Trends Endocrinol Metab.

[38] Takemoto M, Yokote K, Yamazaki M, Ridall AL, Butler WT, Matsumoto T (2000). Enhanced expression of osteopontin by high glucose: involvement of osteopontin in diabetic macroangiopathy. Ann N Y Acad Sci.

[39] Zhang SJ, Wang JP (2013). Effect of pioglitazone on renal osteopontin expression in diabetic rats. Journal of Chinese Pharmaceutical Sciences.

[40] Zhang S, Xu H, Yu X, Wu Y, Sui D (2017). Metformin ameliorates diabetic nephropathy in a rat model of low-dose streptozotocin-induced diabetes. Exp Ther Med.

